# Endometriosis and Medical Therapy: From Progestogens to Progesterone Resistance to GnRH Antagonists: A Review

**DOI:** 10.3390/jcm10051085

**Published:** 2021-03-05

**Authors:** Jacques Donnez, Marie-Madeleine Dolmans

**Affiliations:** 1 Société de Recherche pour l’Infertilité (SRI), 143 Avenue Grandchamp, 1150 Brussels, Belgium; 2 Université Catholique de Louvain, 1200 Brussels, Belgium; 3 Pôle de Recherche en Gynécologie, Institut de Recherche Expérimentale et Clinique, Université Catholique de Louvain, 1200 Brussels, Belgium; marie-madeleine.dolmans@uclouvain.be; 4 Gynecology Department, Cliniques Universitaires Saint Luc, 1200 Brussels, Belgium

**Keywords:** endometriosis, pelvic pain, dysmenorrhea, oral contraceptive pills, progestogens, progesterone resistance, GnRH antagonist, add-back therapy

## Abstract

Background: The first objective of this review was to present, based on recent literature, the most frequently applied medical options (oral contraceptive pills (OCPs) and progestogens) for the management of symptomatic endometriosis, and evaluate their effectiveness in treating premenopausal women with endometriosis-associated pelvic pain, dysmenorrhea, non-menstrual pelvic pain and dyspareunia. The second objective was to review the concept of progesterone resistance and newly available treatment options. Methods: We reviewed the most relevant papers (*n* = 73) on the efficacy of OCPs and progestogens as medical therapy for endometriosis, as well as those on progesterone resistance and new medical alternatives (oral gonadotropin-releasing hormone (GnRH) antagonist). Eleven papers, essentially reviews, were selected and scrutinized from among 94 papers discussing the concept of progesterone resistance. Results: Having reviewed the most significant papers, we can confirm that OCPs and progestogens are effective in two-thirds of women suffering from endometriosis, but that other options are required in case of failure (in one-third of women due to progesterone resistance) or intolerance to these compounds. It is clear that there is a need for effective long-term oral treatment capable of managing endometriosis symptoms, while mitigating the impact of side effects. Biochemical, histological and clinical evidence show that estrogens play a critical role in the pathogenesis of endometriosis, so lowering levels of circulating estrogens should be considered an effective medical approach. The efficacy of three oral GnRH antagonists is discussed on the basis of published studies. Conclusion: There is a place for GnRH antagonists in the management of symptomatic endometriosis and clinical trials should be conducted, taking into account the different phenotypes in order to propose novel algorithms.

## 1. Introduction

The aim of this review is to present, based on recent literature, various medical options for the management of symptomatic endometriosis, a common chronic inflammatory disease causing pain and infertility [1,2,3]. Between 5% and 10% of women of reproductive age are affected [1,3]. There are three distinct forms of the disease (peritoneal, ovarian and rectovaginal endometriosis) and each of them may be associated with specific symptoms, although dysmenorrhea and chronic non-menstrual pelvic pain are the most prevalent [2]. Nevertheless, one common pathogenic mechanism shared by all forms of the condition is the impact of estradiol (E2), which is known to have proinflammatory and antiapoptotic effects on endometrial cells, especially in ectopic foci [2,3].

Based on the role of retrograde menstruation according to Sampson’s theory, blocking ovulation and menstruation by means of hormonal therapies may in theory be disease-modulating and control the symptoms of endometriosis. However, hormonal treatment also impedes conception, excluding this approach in women wishing to conceive [1,4]. Our goal is to analyze various first- and second-line therapies, as well as novel strategies. From a strictly medical and scientific point of view, efficacy, safety and tolerability should be deemed the primary endpoints when contemplating different medical strategies [4,5,6]. Costs cannot be entirely disregarded but should not be the main consideration in the treatment decision.

As reported by Vercellini et al. [5,6], many reviews and systematic analyses of oral contraceptives pills (OCPs) and progestogens for endometriosis have been published, as have guidelines from major gynecological societies (ASRM, ESHRE, ACOG, Canadian Society [7,8,9,10]). Our objective is not to revisit these guidelines, rather to extract the pros and cons from the relevant literature in order to propose new algorithms.

A literature search was conducted through an electronic database (PubMed, Embase) up to December 2020. The following key words were entered: endometriosis, progesterone, progestogen, GnRH agonist, GnRH antagonist, medical therapy, add-back therapy. From 2010 to 2020, 4732 manuscripts reported data and results on medical therapy for endometriosis. The search was limited to peer-reviewed full texts in English, reporting data on medical therapy. After identifying original articles and reviews that were methodologically adequate, very well written, updated, informative and well balanced and taking into account duplicated results and plagiarism, the authors selected and reviewed 73 articles. Ninety-four papers discussed the concept of progesterone resistance and 11 (essentially reviews) were selected and included. Finally, 20 relevant original papers on oral GnRH antagonist in the management of endometriosis were reviewed.

### An Unmet Need

On the one hand, first-line medical therapy (OCPs and progestogens) is effective in two-thirds of women suffering from endometriosis-related pain [5,6]. However, as reported by Surrey et al. [11] and Soliman et al. [12,13], these first-line medical therapies demonstrate limited long-term efficacy, while second-line therapies (injectable depot formulations of gonadotropin-releasing hormone (GnRH) agonists), which are only proposed in case of OCP or progestogen failure, are associated with bothersome menopausal symptoms.

On the other hand, surgery is of course able to eliminate visible endometriotic lesions, but cannot cure the disease. Thus, post-operative recurrence is common, because persistent foci not detected at the time of surgery may progress under the influence of circulating estrogens [1,2,3,5,6]. There is therefore a need for effective long-term oral treatment capable of managing endometriosis symptoms, while alleviating the impact of side effects.

## 2. Recurrent Questions: Are Estroprogestins and Progestins Effective?

### 2.1. Biological Evidence: The Concept of Progesterone Resistance in Endometriosis

#### 2.1.1. Progesterone Receptors and Resistance

During the follicular phase, estrogen acts on eutopic endometrium through the estrogen receptor (ER) to increase transcription and protein levels of the progesterone receptor (PR), especially the PR-B isoform. During the luteal phase, progesterone acts through PR-B and boosts transcription and secretion of 17β-hydroxysteroid dehydrogenase type 2 (17β-HSD2), responsible for converting E2 to the less active estrone [14,15,16].

It was already hypothesized in 1997 that endometrial stromal cells in ectopic endometrium are unable to react to progesterone, as they do in eutopic endometrium, due to the lack of biologically active PRs. In 1997, Nisolle and Donnez [2] reported the absence of secretory transformation, during the luteal phase, in some peritoneal lesions and most deep endometriotic lesions, and different patterns of ERs and PRs in eutopic and ectopic endometrium. This led the authors to hypothesize that PRs, although present, were biologically inactive, suggesting the concept of progesterone resistance. In 2000, Attia et al. reported that PR-B mRNA and protein levels fell in endometriotic lesions, whereas PR-A isoforms were spared, indicating some resistance to progesterone [17]. Since then, numerous papers (more than 90 according to PubMed) have put forward arguments supporting the concept of progesterone resistance. A first review by Bulun et al. suggested that the inability of endometriotic stromal cells to produce progesterone-induced paracrine factors that stimulate 17β-HSD2 may be due to a lack of PR-B, resulting in a deficient E2 metabolism in endometriosis and giving rise to high local levels of E2 [18].

More recently, Bulun et al., reviewing the roles of ERs and PRs in the development of endometriosis, demonstrated that endometriotic stromal cells show significantly lower ERα and higher ERβ levels than eutopic endometrial stromal cells [15]. Indeed, in endometriotic implants, ERα is reduced but ERβ activity is upregulated, leading to complete loss of PR-B, which is then unable to induce 17β-HSD2. ERβ is the key mediator of estrogen action in endometriotic stromal cells and was found to transcriptionally repress ERα, possibly promoting disease progression by several pathways [15,16]. It may interact with the cytoplasmic apoptotic machinery and inflammasome complex to prevent tumor necrosis factor (TNF)-induced cell death and enhance proliferation of endometriotic cells [15,19].

The specific impact of retinoic acid (RA) on progesterone resistance has recently been discussed in several papers [15,20,21,22]. In endometrial stromal cells, progesterone increases formation of RA, which triggers expression of 17β-HSD2 in epithelial cells. Conversely, in endometriotic lesions, stromal cells showing progesterone resistance are unable to produce RA which results in loss of 17β-HSD2 expression and failure to inactivate local E2, ultimately leading to high E2 activity [14,15,16,20,21,22]. As stressed by Pavone et al. [20,21], impaired progesterone action is likely to be the cause of alterations in RA function in endometriotic tissue. RA acts through various RA receptors (RARs) and transcriptional activation of nuclear RARs by RA often generates cell growth inhibition. In normal endometrial cells, RA induces apoptosis via the CRABP2/RAR2 pathway [15,16,18] triggered by PRs. In contrast, PR resistance in endometriotic cells results in CRABP2 deficiency, thereby favoring survival, development and persistence of endometriotic implants [15,16].

#### 2.1.2. Causes of Progesterone Resistance

The causes of progesterone resistance in adult women were reviewed by Patel et al. [22] and Bulun et al. [15], and the most important are summarized and updated in this manuscript.

#### Congenital

According to Gargett et al. [23], endometriosis and progesterone resistance observed in adult women may be a consequence of neonatal progesterone resistance persisting through early adolescence. By immuno-histochemical analysis of neonatal endometrial biopsies, partial or complete progesterone resistance was identified in over 66% of cases in neonatal endometrium [24], while a full response to progesterone was observed in around 33% [24]. It should be noted that newborn menstruation resulting from postpartum withdrawal of placental steroid hormones, ranging from 3% to 6%, may be responsible for establishment of endometriosis because of occlusion of the cervical canal by dense cervical secretions [25,26].

#### Inflammation and Oxidative Stress

Retrograde menstruation and bleeding of endometriotic lesions are responsible for the presence of erythrocytes in the pelvic cavity, which are likely to release pro-oxidant and proinflammatory factors like hemoglobin and its highly toxic byproducts heme and iron [27,28,29,30]. As reviewed by Donnez et al. [30], erythrocytes, apoptotic endometrial tissue and cell debris transplanted inside the peritoneal cavity are potential inducers of oxidative stress. The above-mentioned pro-oxidant and proinflammatory factors and toxic byproducts both play a key role in the formation of deleterious reactive oxygen species (ROS) [30,31]. It was suggested that detoxifying systems, while present, might be insufficient to correctly metabolize hemoglobin in the context of active endometriosis [28,29,30]. Indeed, in some patients, peritoneal protective mechanisms are swamped by menstrual blood, either due to an abundance of menstrual reflux or because scavenging systems are defective [27,28,29,30]. Iron overload, oxidative stress and inflammation could all be the consequence of lysis of erythrocytes [27]. Cellular iron storage within ferritin in macrophages may limit its toxicity, but continuous delivery of iron to macrophages might overwhelm the capacity of ferritin to store iron, inducing oxidative stress [29,32].

Progesterone has anti-inflammatory properties in uterine cells [32], but endometriosis is an inflammatory disease [29]. Proinflammatory factors (like heme and iron) and cytokines (like tumor necrosis factor-alpha (TNFα) and interleukin 1 beta (IL1β)) may also disrupt PR function and play a role in progesterone resistance which finally leads to increased nuclear factor kappa B (NF-kB) activity, which is involved in endometriosis development [27,33,34].

#### Genetics and Epigenetics

Evidence for a genetic basis for endometriosis has been reported in several studies [35], with a number of polymorphisms in the PR gene implicated as a genetic cause of progesterone resistance. The genetic-epigenetic theory was recently developed by Koninckx et al. [36], and genetic and epigenetic links were also discussed in a very recent review by Zubrzyeka et al. [37]. Accumulating evidence supports the concept of epigenetic influence, along with family predisposition and genetic causes. This epigenetic information is reflected in levels of DNA methylation, histone modifications and microRNA (miRNA) expression [15,22,33,37,38].

Wu et al. [39] first reported hypermethylation and silencing of the homeobox A 10 (HOXA-10) promoter. In eutopic endometrial cells of endometriosis patients, HOXA-10 does not show increased expression after ovulation due to hypermethylation and gene silencing of its promoter [39]. Furthermore, these endometrial cells also exhibit hypermethylation of the PR-B promoter, inducing expression of the receptor protein [38,39,40] and finally resulting in a state of progesterone resistance. Epigenetic changes to the PR promoter could be attributed to TCDD dioxin (2,3,7,8 tetrachlorodi-benzo-p-dioxin) exposure, which plays a role in inflammation and oxidative stress too [22,41,42,43].

Epigenetic regulation of endometriosis also includes alterations to miRNAs, which are non-coding RNA fragments that inhibit protein expression and cause mRNA degradation. As reviewed by Patel et al. [22], some miRNAs (miR-9m, miR-34 and miR-29c) are implicated in progesterone resistance.

#### Mesenchymal Progenitors

Barragan et al. [44] suggested that human endometrial fibroblasts derived from mesenchymal progenitors (mesenchymal stem cells (MSCs)) inherit progesterone resistance and acquire an inflammatory phenotype in the endometrial niche in endometriosis. Indeed, by analyzing transcriptomes of eutopic endometrial MSCs (eMSCs) and endometrial stromal fibroblasts (eSFs) after fluorescence-activated cell sorting (FACS), these authors confirmed that eMSCs are progenitors of eSFs, and that eSFs in endometriosis show progesterone resistance inherited from eMSCs.

#### Phenotype of Endometriosis

The three different entities of endometriosis (peritoneal, ovarian and deep nodular endometriosis) were described in 1997 [2]. Peritoneal lesions were also classified into red, black and white lesions [2] and included in the revised ASRM classification [45]. Red lesions were found to be very active, with high proliferative activity and vascular endothelial growth factor (VEGF) and matrix metalloproteinase (MMP) content, explaining their frequent bleeding and remodeling during the menstrual period [46,47,48]. Active endometriotic lesions are highly infiltrated by macrophages that are present in the stroma and play a role in the development of the disease [49,50]. These red lesions are resistant to progestin therapy, which may induce some decidualization, but not atrophy [46,47,48]. In a review by Redwine [51], numerous differences were highlighted between eutopic and ectopic endometrium, possibly explaining not only the heterogeneity of lesions, but also their intravariability when luteal phase-induced changes are analyzed.

It has been known for a long time that the response of deep endometriotic nodules to medical therapy (OCPs or progestogens) is a source of controversy. Some authors [52] favor progestogens, while others find them relatively ineffective [1,2,53,54]. As demonstrated in 1995, a progestogen (lynestrenol) was unable to reduce vascularization (evaluated by the capillary–stroma relative surface area) in endometriotic lesions, but a significant decrease was observed after GnRH agonist therapy [55]. A recent review by Reis et al. [56] also confirms that deep endometriosis appears to be more resistant to regression upon medical treatment. According to the literature, some authors found a substantial volume reduction during OCP and norethisterone acetate (NETA) therapy [57], but this was not confirmed by other more recent studies [53,56]. Indeed, PRs may be present, but biologically inactive. On the other hand, PRs may well be absent, leading to progesterone resistance and no decrease in lesion size with OCP or progestogen therapy [58].

### 2.2. Clinical Evidence: Estroprogestins and Progestogens

#### 2.2.1. Estroprogestins: OCPs

Use of OCPs containing estroprogestins is considered off-label, despite being included in guidelines on endometriosis management issued by various authoritative societies. Vercellini et al. [5,6,59,60,61,62] advocate use of estroprogestins for the treatment of endometriosis but, as acknowledged by the authors themselves, 33% of patients given estroprogestins and/or progestins do not respond to therapy. Progesterone resistance may of course explain this unexpected issue [18,19,20,21,22]. In one of their numerous reviews, Vercellini et al. [5] concluded that estroprogestins containing the lowest possible dose of ethinyl estradiol (EE) and second-generation progestins should be used as first-line treatment in low- and intermediate-risk cases. They clearly established that the value of therapy depends on a balance between potential benefits, possible harms, and cost of care [5,61,62].

However, this raises an important question, namely, what is the impact of continuous rather than cyclic OCP use in terms of efficacy and safety? With continuous OCP administration, dysmenorrhea may be reduced compared to cyclic use, but the incidence of erratic bleeding may increase, and safety issues have not been fully resolved. In fact, individual patient preferences should govern choice, as some women prefer an absence of menstrual bleeding, while others consider amenorrhea a non-physiological state and prefer to experience some bleeding at the time of menstruation.

#### Pros and Cons of OCPs

In recent reviews [59,60,61,62], Vercellini et al. carefully evaluated the pros and cons of OCPs. By reducing the volume of withdrawal bleeding secondary to minimal endometrial growth and mitigating retrograde blood flow and hence pelvic oxidative stress, these authors concluded that OCPs can play an important role in the management of endometriosis, as two-thirds of women with symptomatic endometriosis respond to estroprogestins.

Surprisingly, only one randomized placebo-controlled clinical trial of OCPs in endometriosis has ever been published [63]. A statistically significant, though modest, improvement in dysmenorrhea was found with OCPs given for four months compared to placebo. OCP administration resulted in about a 50% reduction in dysmenorrhea. There was, however, a lack of any beneficial effect of OCPs on non-menstrual pelvic pain and dyspareunia, and these studies failed to report data on their efficacy according to lesion phenotype [59,60,61,62]

Safety nevertheless remains a concern, as patients may need long-term periods of therapy during their advanced reproductive years, and the risk of venous or arterial thrombosis is not negligible [54,55,56,57,58,59,60,61,62,63,64,65,66,67]. The relative risk (RR) is dependent on the type of progestins used for estroprogestin preparation, but progestin-only preparations (NETA and desogestrel pills) were not shown to increase the risk of venous thromboembolism [67]. Despite being 3 to 4 times more frequent, venous thrombotic events are much less severe than arterial thrombotic events, which are more associated with the EE dose than with the progestin type [67]. Safety aspects are of utmost importance considering that many women may require treatment for several years [68].

In addition, Brion et al. [69] and Speroff et al. [70], reported that 5 µg EE is equivalent to about 1 mg micronized E2 and, as stressed by Casper [68], a dose of 20 µg EE in low-dose OCPs is equivalent to 4–6 times the physiological dose of estrogen, which may promote attachment of endometrial cells deposited in the pelvis. One study found that 70% of women had used multiple OCPs for endometriosis, and over 40% had been prescribed between 3 and 10 different OCPs for symptom control [71]. According to Casper (68), these data suggest that patients experiencing recurrence of pelvic pain while taking an OCP needed to be switched to a different OCP, supporting the notion that OCPs are not completely effective for treatment of endometriosis. Moreover, a number of studies suggest an adverse effect of OCPs on the incidence of endometriosis. Indeed, an increased risk of the disease was observed in past users of OCPs [72,73], reinforcing the hypothesis that high doses of estrogen in OCPs could lead to progression to a more invasive type of endometriosis [68,72].

In a recent overview of Cochrane reviews, Brown and Farquhar [74] concluded that while OCPs are widely used to treat endometriosis-related pain, evidence of their efficacy is limited and there are insufficient data to make any judgment in terms of comparisons with other medical therapies. In a systematic review of the evidence, Jensen et al. [75] found that the available literature considers combined hormone contraceptives to be effective for relief of endometriosis-related pain. However, corroborative data are of low quality and inadequate to draw conclusions on the superiority and relative benefits compared to other approaches [75].

Moreover, due to progesterone resistance, the progestin contained within the OCP and designed to antagonize the estrogen effect sometimes fails to produce the desired effect in endometriotic implants, resulting in relative estrogen dominance.

#### 2.2.2. Progestins

Progestins are synthetic compounds that reduce the frequency of pulsatile GnRH release, leading to lower pituitary secretion of follicle-stimulating hormone (FSH) and luteinizing hormone (LH), and ultimately suppression of ovarian steroid secretion.

Buggio et al. [76] wrote an excellent review on available progestins adopted in the management of endometriosis which includes a wide range of both oral and depot compounds (NETA, dioenogest, desorgestrel, cyproterone acetate, depot medroxyprogesterone acetate (DMPA), levonorgestrel-releasing intrauterine system (LNG-IUS), etonorgestrel subdermal implant). They concluded that all available progestins are effective in controlling pain symptoms in two-thirds of women with endometriosis. As there are not enough robust data demonstrating the superiority of one progestin over the others, they also concluded that oral norethisterone acetate (NETA) should be considered the first choice, given the extremely favorable cost-effectiveness profile.

In a recent review, Reis et al. [56] summarized the effect of progestins on endometriotic lesions as generating different levels of downregulation of ERs, a drop in local E2 production, a reduction in inflammation, and creation of a pseudo-pregnancy condition. However, the impact of progestogens on the morphology and vascularization of ectopic endometrial tissue and atrophy is much more questionable [77,78,79,80,81]. Effects in terms of atrophy are much less pronounced than with GnRh agonist [81]. Some studies have revealed the absence of proliferation index changes under progestogen therapy [77,78,79,80,81]. A recent study demonstrated no differences in ovarian endometriomas in terms of necrosis or glandular atrophy between women receiving dienogest and those not [82].

Casper [68] strongly asserts that progestin-only pills constitute a better first-line approach than estroprogestins but, according to Vercellini et al. [59,60,61,62], progestin-only therapy should be reserved for high-risk women (with deep endometriosis), or those with contraindications or intolerance to estroprogestins. Among available progestins, NETA should be considered the drug of choice given its extremely favorable cost-effectiveness profile and the possibility of switching to dienogest (in case of poor response to NETA) [59].

A study comparing NETA and dienogest [83] reported that 70% and 72% of patients, respectively, were very satisfied or satisfied, meaning that a substantial proportion, around 30% of women, were left dissatisfied, 10% of whom were very dissatisfied. Efficacy was similar in terms of pain reduction (dysmenorrhea, dyspareunia, non-menstrual pelvic pain). It was noted that menstrual pain (dysmenorrhea) was almost eradicated in both groups as a result of amenorrhea induced by the two progestins [83]. Of course, suppressing ovulation and reducing the amount of menstrual bleeding greatly reduces the number of erythrocytes regurgitated into the pelvis, resulting in a decrease in oxidative stress and the inflammatory environment [30]. However, as mentioned earlier, endometriotic stromal cells are poorly differentiated with deficient PRs and fail to send physiological paracrine signals to adjacent epithelial cells, explaining the absence of response in more than 30% of women treated with progestins [58,84].

#### 2.2.3. Summary

PR deficiency is evident in endometriotic lesions, leading to progesterone resistance and defective progesterone action, with obvious consequences on the survival and development of endometriotic tissues [15,22,84]. Moreover, as proved by a recent study [58], PR status is strongly associated with a response to progestin-based therapies (including combined OCPs). Indeed, in this study, low PR levels were observed in subjects who failed to respond.

## 3. Why Do We Need New Options?

New options are needed because of concerns about estroprogestins and progestin-only drugs [85], namely:

- Two-thirds of symptomatic women find pain relief and improvement in their general condition thanks to estroprogestins and progestin-only medication, but one-third are non-responders due to progesterone resistance [5,6,61,62,68].
- There is an increased risk of venous or arterial embolism [65,66,67].
- The side effects of estroprogestins are essentially related to the type of progestin used [67].
- The reduction in lesion volume is not predictable and not significant in the vast majority of cases. Conflicting results have been observed [77,78,79,80,81,82,86,87,88].

Moreover, among new drugs, selective progesterone receptor modulators (SPRMs) are not an option as they induce progesterone modulator-associated endometrial changes (PAECs) in ectopic foci and their efficacy is limited [89,90,91]. In addition, randomized controlled trials (RCTs) to evaluate the effect of SPRMs on endometriosis are lacking.

### 3.1. The Optimal Goal of Medical Therapy

As suggested by Barbieri in his threshold hypothesis [92], the ideal solution would be to lower E2 levels enough to induce amenorrhea and treat symptoms, while maintaining sufficient levels to mitigate severe side effects, such as vasomotor menopausal symptoms (essentially, hot flushes) and bone mineral density (BMD) loss. Partial suppression of E2 within the 30–60 pg/mL range could be the optimal compromise between efficacy, tolerance and safety [85].

#### How Do We Achieve Partial E2 Suppression?

To date, the only option to restore sufficient E2 levels to avoid menopausal symptoms and BMD loss has been combined administration of a GnRH agonist (depot injection) and estrogens/progestins (add-back therapy). Based on results from RCTs in women with endometriosis, NETA (5 mg/d) was approved by the Food and Drug Administration (FDA) as add-back therapy when combined with a GnRH agonist [93]. On the other hand, according to ESHRE guidelines, progestogen-only add-back therapy does not prevent BMD loss [94].

GnRH agonists are effective at treating endometriosis symptoms, but they have numerous limitations, including a delayed therapeutic impact because of the flare-up effect, excessive suppression of E2 to less than 20 pg/mL (with related unfavorable side effects), inability to titrate E2 levels, and unpredictable reversibility of treatment when injectable depot forms of GnRH agonists are used [95,96,97,98].

### 3.2. GnRH Antagonist: The Ideal New Option?

**Figure 1:**
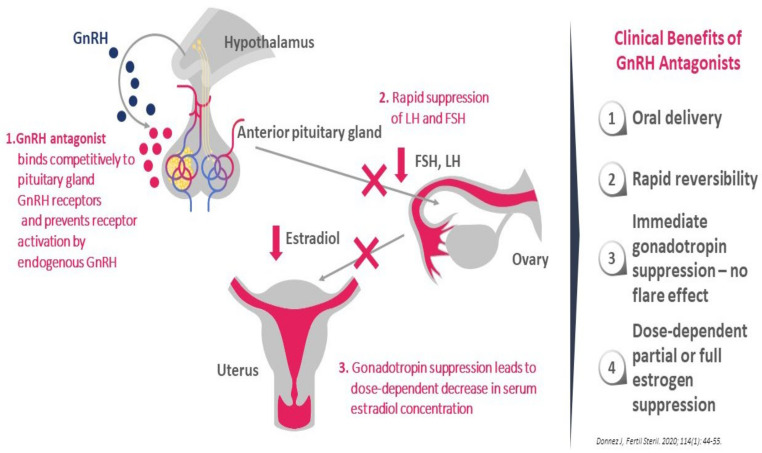
GnRH antagonist mechanism of action.

Recently, emerging use of GnRH antagonist has been the focus of several papers [99,100,101,102,103,104,105,106,107,108]. These drugs cause competitive blockage of the GnRH receptor and hence dose-dependently suppress production of FSH and LH and inhibit secretion of ovarian steroid hormones without inducing a flare-up effect (Figure 1). The mechanism is different from that of the GnRH agonist which, after a first phase of stimulation, desensitizes GnRH receptors, leading to full suppression of LH and FSH production and subsequently to complete suppression of E2 to levels similar to those observed after bilateral oophorectomy [95,96,97,98].

**Figure 2:**
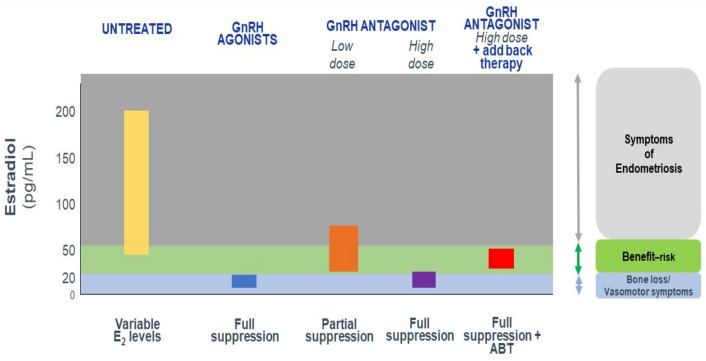
Expected estradiol (E2) levels during the menstrual cycle, under GnRH agonist and under GnRH antagonist therapy without and with add-back therapy (ABT).

The main advantages of GnRH antagonists are [85,99,100,101,102,103,104,105,106,107,108,109]:

1. They produce dose-dependent estrogen suppression, from partial suppression at lower doses to almost full suppression at higher doses (Figure 2).
2. There is rapid reversibility and recovery of hormone secretion after stopping treatment.
3. Striking a balance between efficacy and safety/tolerability may unlock the potential of this new class of drug, suggesting the possibility of individual tailoring according to symptoms and the wishes of the patient.
4. As estrogens play a crucial role not only in survival of endometriotic implants, but also in coordinated vasculogenesis and neurogenesis [110] by different pathways involving ER signaling [15,16], it is logical to consider lowering estrogen levels as a therapeutic approach.

One GnRH antagonist (elagolix) was approved by the US FDA in July 2018 [109]. Two more oral GnRH antagonists, namely, relugolix (TAK385) [104] and linzagolix (OBE-2109) [105], have recently yielded very robust results in randomized, placebo-controlled clinical trials for the treatment of pain associated with endometriosis, and will be discussed in detail further. An improvement in symptoms, mainly pain resulting from inflammatory endometriotic lesions, should be the main goal of long-term treatment. By inducing amenorrhea and halting menstrual bleeding and reflux, or even simply lessening its severity, pelvic oxidative stress, the main source of inflammation, can be significantly reduced [30].

### 3.3. Elagolix

Elagolix is rapidly absorbed after oral administration and its mean plasma half-life (t1/2) ranges from 2.4 to 6.3 h [99,107,110]. It is mainly metabolized by the liver (mostly cytochrome P450 3A (CYP3A)-mediated), and 90% of excretion of elagolix and its metabolites occurs in the feces [99]. Overall, the pharmacokinetic properties of elagolix at 150 mg once daily and 200 mg twice daily do not appear to be affected, to a clinically meaningful extent, by body weight, body mass index, race/ethnicity or the presence of endometriosis [102,103,107].

#### Clinical Efficacy

The efficacy of six-month treatment with elagolix was evaluated in two large, double-blind, phase III trials (Elaris EM-I and Elaris EM-II) [102]. Overall, 872 and 817 women with pain and moderate-to-severe endometriosis underwent randomization in Elaris EM-I and EM-II, respectively. Two different regimens of elagolix (150 mg once daily and 200 mg twice daily) were tested.

The two co-primary efficacy endpoints were the proportion of women who showed clinically meaningful responses with respect to dysmenorrhea and non-menstrual pelvic pain at three months of treatment. The percentage of women who experienced a clinical improvement in dysmenorrhea was 46.4% with elagolix 150 mg once daily and 75.8% with elagolix 200 mg twice daily (compared with 19.6% in the placebo group). In Elaris EM-II, corresponding percentages were 43.4% and 72.4% (compared with 22.7% in the placebo group). In Elaris EM-I, the percentage of women who noted an improvement in non-menstrual pelvic pain was 50.4% with elagolix 150 mg once daily and 54.5% with elagolix 200 mg twice daily (compared with 36.5% in the placebo group). In Elaris EM-II, corresponding percentages were 49.8% and 57.8% (compared with 36.5% in the placebo group). Overall, alleviation of both dysmenorrhea and non-menstrual pelvic pain were sustained for six months (Table 1). In both studies, only women treated with elagolix at a dose of 200 mg twice daily showed a statistically significant greater reduction in dyspareunia after three months of treatment (compared with the placebo).

Achieving a clinically meaningful response to endometriosis-related pain with elagolix was found to be associated with improvements in health-related quality of life and work productivity [111,112]. Accordingly, significantly more women taking either dose of elagolix reported “much” or “very much” improvement on the patient global impression of change (PGIC) scale and a better quality of life on the EHP-30 questionnaire compared to women taking the placebo.

**Figure 3:**
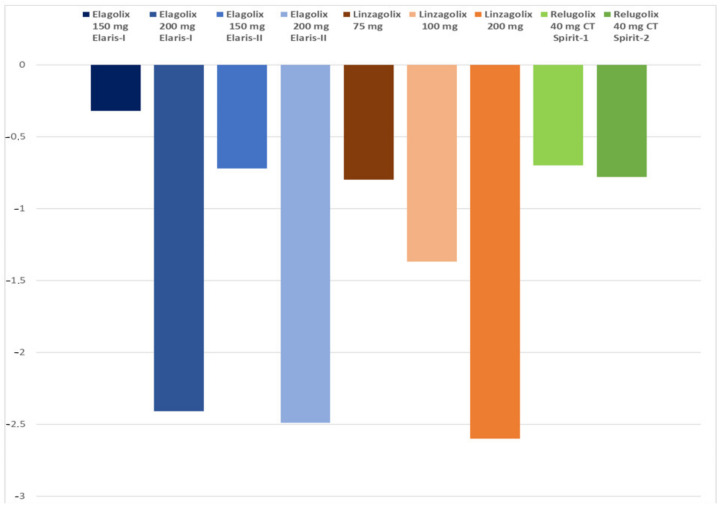
Mean percentage of bone mineral density (BMD) loss at week 24 (lumbar spine) in the different groups of women treated by different doses of GnRH antagonist (elagolix 150 mg once daily; elagolix 200 mg twice daily; linzagolix 75 mg, 100 mg and 200 mg once daily; and relugolix 40 mg combination therapy (CT): relugolix plus add-back therapy, once daily).

Elagolix causes a dose-dependent decrease in BMD. In the EM-I and -II trials, Taylor et al. [102] reported that mean decreases in BMD in the lumbar spine, femoral neck and total hip after six months of treatment were significantly greater in patients receiving elagolix than in those given a placebo (excluding femoral neck BMD at a dose of 150 mg in Elaris EM-I (Figure 3). The percentage of patients with a decrease of >5% from baseline in lumbar spine BMD was higher in the elagolix 200 mg twice daily group in all studies. It should be noted that decreased BMD was the most common cause of treatment discontinuation in subsequent Elaris EM-III and -IV studies described below.

In phase III extension studies (Elaris EM-III and EM-IV) [103], 569 women (44.3% of those who had concluded the EM-I and EM-II trials) continued to receive elagolix for 6 additional months, with post-treatment follow-up of up to 12 months. The majority of women reported much or very much improved endometriosis-associated pain after 12 months of treatment. Specifically, upon completion of treatment, respective responder rates for dysmenorrhea in Elaris EM-III and EM-IV were 52.1% and 50.8% with elagolix 150 mg once daily, and 78.1% and 75.9% with 200 mg twice daily (Table 2). Responder rates for non-menstrual pelvic pain were 67.8% and 66.4% with elagolix 150 mg once daily, and 69.1% and 67.2% with 200 mg twice daily. Elagolix did not completely suppress ovulation at either of the two doses.

Regarding side effects, the most frequently reported were hot flushes, depending on the dose. Percentages of women experiencing hot flushes at a dose of 150 mg (once daily) were respectively 23.7%, 22.6%, 44% and 36% in Elaris-I, -II, -III and -IV. At 200 mg (twice daily), percentages were respectively 42.3%, 47.6%, 72% and 77% [102,103].

**Figure 4:**
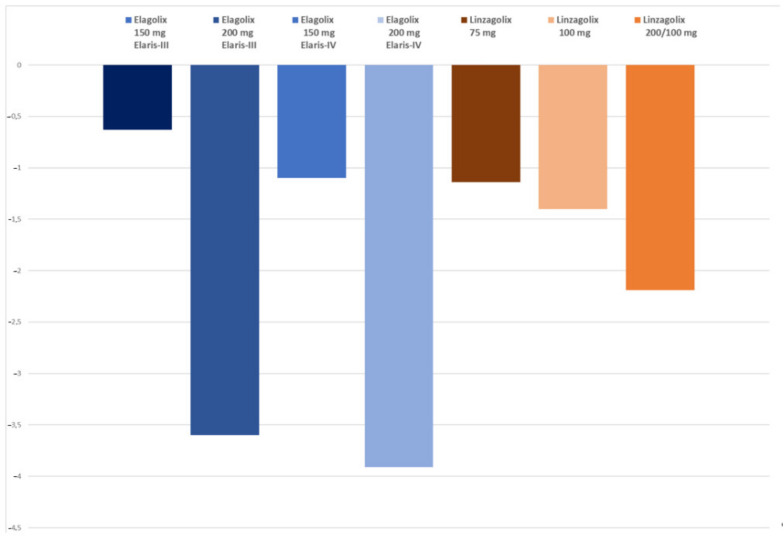
Mean percentage of BMD loss at week 52 (lumbar spine) in the different group of women treated by different doses of GnRH antagonist (elagolix and linzagolix). Patients randomized to linzagolix 200 mg were switched to linzagolix 100 mg at week 24.

In conclusion, use of elagolix 200 mg twice daily causes strong suppression of E2 and marked improvements in dysmenorrhea, non-menstrual pelvic pain and dyspareunia, albeit at the cost of more hot flushes and a more pronounced decrease in BMD (Figure 4). As reported by Taylor et al. [107], the recommended treatment duration in women with normal liver function or mild hepatic impairment is up to 24 months with elagolix 150 mg once daily, and up to 6 months with 200 mg twice daily.

Current research is focused on determining the impact of add-back therapy during treatment with elagolix. Two ongoing randomized, parallel-assignment, phase III studies are evaluating the effectiveness and safety of 24 months of treatment with elagolix alone or elagolix plus E2/NETA (as add-back therapy) in women with a diagnosis of endometriosis experiencing moderate-to-severe endometriosis-associated pain [107].

### 3.4. Linzagolix

Linzagolix has a half-life of 15–18 h, high oral bioavailability, low volume of distribution, no food effects and an absence of interactions with transporters and CYP3A4 enzymes [98,100]. In a recent paper, Donnez et al. [105] evaluated the impact of different doses of linzagolix, a new oral GnRH antagonist administered once daily for 24 weeks (50 mg, 75 mg, 100 mg and 200 mg), on endometriosis-associated pain in a series of 328 patients. We will be focusing on the three doses that will be promoted by the company (75 mg, 100 mg and 200 mg). Doses of 75 mg without add-back therapy and 200 mg with add-back therapy are currently being investigated in phase III endometriosis clinical trials.

#### Clinical Efficacy

Percentages of women with a ≥30% reduction in overall pelvic pain (primary efficacy endpoint) at week 12 were respectively 61.5%, 56.4% and 56.3% in the 75 mg, 100 mg and 200 mg groups compared to 34.5% in the placebo group [105]. A significant reduction in overall pelvic pain was already evident four to eight weeks after starting treatment. Percentages of women with a ≥30% reduction in overall pelvic pain at week 24 were 70.8%, 66.7% and 77.3% in the 75 mg, 100 mg and 200 mg groups, respectively, in phase II trials (Table 1).

A similar pattern was observed in responder rates for dysmenorrhea and non-menstrual pelvic pain, with significant differences observed compared to the placebo at 12 weeks with doses of 75 mg, 100 mg and 200 mg. Percentages of women experiencing a reduction of ≥30% in both dysmenorrhea and non-menstrual pelvic pain at 12 weeks were respectively 68.2% and 58.5% in the 75 mg group, 68.6% and 61.5% in the 100 mg group and 78.9% and 47.7% in the 200 mg group. Response rates for overall pelvic pain, dysmenorrhea and non-menstrual pelvic pain were maintained or increased after 24 weeks of treatment. Percentages of women experiencing a reduction of >30% in both dysmenorrhea and non-menstrual pelvic pain at week 24 were 58.3% and 72.9% in the 75 mg group, 82.1% and 64.1% in the 100 mg group and 84.1% and 72.7% in the 200 mg group. Compared to the placebo, there was a significant reduction in dyspareunia at 12 weeks with the 200 mg dose, but not with 75 mg or 100 mg. This was also encountered with another GnRH antagonist, elagolix. Indeed, dyspareunia was only significantly alleviated at the highest daily dose (200 mg twice daily), but not at a lower dose (150 mg once daily), while other types of endometriosis-associated pain were significantly reduced with both the lower and higher dose [96]. It is not clear why dyspareunia appears to respond differently to treatment with a GnRH antagonist compared to other types of endometriosis-associated pain (105).

**Figure 5:**
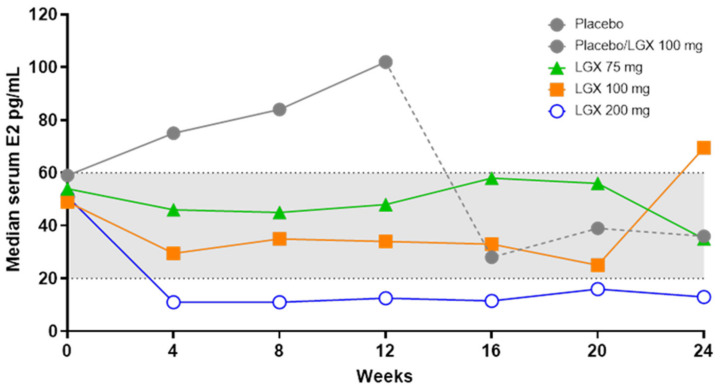
E2 level up to week 24 in women under placebo, linzagolix (LGX) 75 mg, 100 mg and 200 mg. Patients under placebo were switched to linzagolix 100 mg at week 12.

There was a dose-dependent increase in the rate of amenorrhea from weeks 4 to 24. At week 12, estimated amenorrhea rates were 4.3% in the placebo group and 36.3%, 55.8% and 80.9% in the 75 mg, 100 mg and 200 mg dose groups, respectively. Concerning serum E2, there was rapid and full suppression to 11 pg/mL in the 200 mg dose group by week 4, which was maintained (range 11–16 pg/mL) up to week 24 (Figure 5). With the 75 mg and 100 mg doses of linzagolix, there was dose-dependent partial suppression of serum E2 to between 20 and 60 pg/mL.

Significantly more women reported “much” or “very much” improvement on the PGIC scale by week 12 in the 75 mg, 100 mg and 200 mg dose groups compared to the placebo group. Based on EHP-30 questionnaire results, treatment with linzagolix resulted in enhanced quality of life. Concerning the different parts of the EHP-30 questionnaire, pain and powerlessness domains were significantly transformed with 75 mg, 100 mg and 200 mg doses, whereas for other domains linked to emotional well-being, social support and self-image, there was a consistent significant difference only in the 200 mg dose group. These results suggest that although partial suppression of E2 has a significant impact on important aspects of general well-being, full suppression with 200 mg has a more constant effect across more aspects of quality of life affected by endometriosis [105].

Mean percentage (95% CI) BMD changes for the lumbar spine from baseline to week 24 in the 75 mg, 100 mg and 200 mg dose groups were −0.80% (−1.57, −0.03), −1.37% (−2.14, −0.59) and −2.60% (−3.56, −1.65), respectively. Most subjects taking the 200 mg dose showed a BMD decrease of >3% at 24 weeks, which would necessitate hormone add-back therapy for longer-term use. It should be noted that calcium and vitamin D supplementation were not provided as part of the trial protocol [105].

Hot flushes were more frequent at higher doses of linzagolix, namely, 19.3%, 26.9% and 42.1% with 75 mg, 100 mg and 200 mg, respectively, at week 12, with similar dose-related differences at week 24 (Table 1) [105].

Results of long-term therapy (52 weeks) were presented at the ASRM and ACOG meetings [113,114]. Response rates for overall pelvic pain, dysmenorrhea and non-menstrual pelvic pain were maintained or increased after 52 weeks of treatment. Patients randomized to linzagolix 200 mg were switched to linzagolix 100 mg at week 24. Percentages of women with a ≥30% reduction in overall pelvic pain (OPP) (primary efficacy endpoint) at week 52 were 69.2%, 53.8% and 82.4% in the 75 mg, 100 mg and 200/100 mg groups, respectively. Percentages of women experiencing a reduction of ≥30% in both dysmenorrhea and non-menstrual pelvic pain at week 52 were respectively 69.2% and 69.2% in the 75 mg group, 69.2% and 53.8% in the 100 mg group and 64.7 % and 76.5% in the 200/100 mg group. Mean percentage (95% CI) BMD changes in the lumbar spine were −1.14 (−2.21 −0.07) at a dose of 75 mg, −1.40 (−3.35 +0.55) at a dose of 100 mg and −2.19 (−3.59 −0.78) at a dose of 200/100 mg.

A recent paper demonstrated the efficacy of linzagolix 200 mg in reducing lesion size and improving quality of life in case of severe adenomyosis, which is often associated with deep endometriosis [108,115]. Borini et al. [116] suggested linzagolix as a possible treatment for assisted reproduction patients presenting with concurrent endometriosis and adenomyosis.

In conclusion, consistent with full suppression of serum E2 to postmenopausal levels, once-daily linzagolix 200 mg provided an additional significant impact on dyspareunia and some aspects of quality of life. However, higher rates of hypoestrogenic symptoms were observed, including BMD loss of ≥3% after 24 weeks, indicating that this once-daily dose will require hormone add-back therapy if used for longer than six months [105].

### 3.5. Relugolix

Relugolix has a half-life of 37–42 h and is mainly metabolized by the liver [104,117]. A phase II, open-label RCT including 487 women with endometriosis-associated pain found relugolix (10 mg, 20 mg or 40 mg orally once daily) and leuprolin for 24 weeks to be equally effective [104]. At the 40 mg dose, hot flushes showed a similar frequency to the leuprolin group.

#### Clinical Efficacy

**Figure 6:**
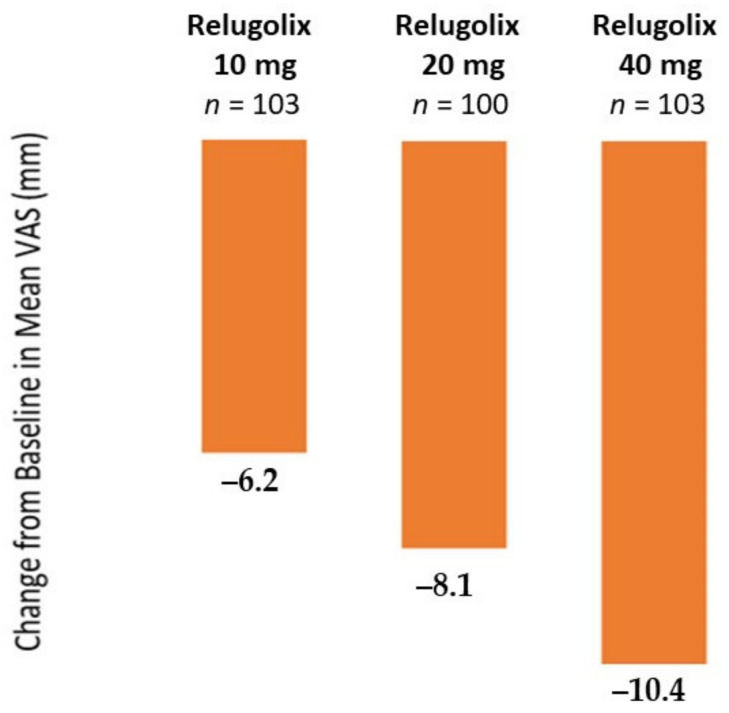
Mean change from baseline in the visual analog scale (VAS) score for pelvic pain for 28 days before the end of treatment period (adapted from Osuga et al. [104]).

Regarding the primary endpoint of the phase II trial, changes from baseline in the mean visual analog scale (VAS) score for pelvic pain were −6.2 in the relugolix 10 mg group and −8.2 in the relugolix 20 mg group (Figure 6). Change was −10.4 in the relugolix 40 mg group, similar to observations in the leuprolin group [104]. Concerning the secondary endpoint, mean VAS scores for pelvic pain and dysmenorrhea started to fall within the first month of treatment, but results for dyspareunia showed no clear trend with relugolix. For additional endpoints, findings similar to the VAS score were obtained with other indices for pain symptoms, such as the Biberoglu and Behrman (B&B) scale [118]. In terms of quality of life, the EHP-30 score was better in relugolix-treated patients than in the placebo group. E2 and FSH levels decreased more rapidly after starting treatment with relugolix 40 mg than with leuprolin. Mean percentage changes in BMD from baseline were −2.1% in the relugolix 40 mg group and −2.2% in the leuprolin group [104].

Results of phase III clinical trials on endometriosis were presented at the last ASRM meeting [119,120]. Two phase III clinical trials were conducted, SPIRIT-1 (*n* = 635) and SPIRIT-2 (*n* = 616), to evaluate the efficacy and safety of the medication (Table 1). Co-primary endpoints at week 24 were assessed by the numerical rating scale (NRS). Clinical responders were defined as those achieving a mean reduction in NRS scores of ≥2.8 points for dysmenorrhea or ≥2.1 points for non-menstrual pelvic pain, with no increase in use of analgesic medications recorded in a daily e-diary at week 24 or at the end of the pain treatment assessment period. In both of these phase III clinical trials, three arms were proposed: placebo, relugolix 40 mg and relugolix combined therapy (CT) (relugolix 40 mg plus 1 mg E2 and 0.5 mg NETA as add-back therapy). After 12 weeks, patients who received 40 mg relugolix alone were switched to the combined arm (relugolix CT).

A similar pattern was observed in responder rates for dysmenorrhea and non-menstrual pelvic pain, with significant differences compared to the placebo at 12 weeks in the relugolix CT arm in both SPIRIT-1 and -2. Response rates for dysmenorrhea and non-menstrual pelvic pain were maintained or increased after 24 weeks of treatment. Percentages of women showing a mean reduction in NRS scores of ≥2.8 points for dysmenorrhea and ≥2.1 points for non-menstrual pelvic pain at week 24 were respectively 75.5% and 58.5% in the relugolix CT arm in SPIRIT-1, and 75.2% and 66% in the relugolix CT arm in SPIRIT-2. All results were statistically significant compared to the placebo (*p* < 0.0001). Dysmenorrhea decreased rapidly from severe at baseline to mild by week 8 and was sustained through to week 24 in 73% and 75% of women in SPIRIT-1 and -2, respectively. Relugolix CT yielded significant improvements in dyspareunia, with 40% and 46% reductions from baseline in NRS scores (mean change). These findings were also statistically significant in SPIRIT-1 and -2 (*p* = 0.0149 and *p* = 0.0371, respectively). Relugolix CT enhanced daily functioning, as demonstrated by the EHP-30 pain score domain at week 24 (*p* < 0.0001 in both studies) Mean percentage BMD changes in the lumbar spine from baseline to week 24 in the relugolix CT and delayed relugolix CT arms were respectively −0.70 % and −1.99% in SPIRIT-1 and −0.78% and −1.92% in SPIRIT-2. Mean percentage hot flushes in the relugolix CT and delayed relugolix CT groups at week 24 were 10.4% and 33.6% in SPIRIT-1 and 13.6% and 35% in SPIRIT-2 [118,119].

In conclusion, oral relugolix CT taken once daily significantly reduced dysmenorrhea and non-menstrual pelvic pain in women with endometriosis compared to a placebo. Relugolix CT is well tolerated, the incidence of vasomotor symptoms is similar to the placebo, and BMD is maintained over 24 weeks.

## 4. Discussion and Conclusion: A Combined Symptom-Oriented and Phenotype-Adapted Approach

According to the ASRM Practice Committee [121], endometriosis requires a life-long management plan, with the goal of maximizing use of medical therapy and avoiding repeated surgical procedures. Following the first publication in the NEJM by Taylor et al. [102] on the impact of elagolix on endometriosis-associated pain and approval from the FDA, Vercellini et al. [122] concluded, in a paper entitled “All that glitters is not gold”, that the efficacy of GnRH antagonist should be proved by pragmatic trials comparing elagolix with low-dose hormone contraceptives and progestogens. The authors argued that trade-offs between health outcomes and costs need to be carefully weighed up, and proposed a stepwise approach starting with OCPs or low-cost progestogens and moving up to high-cost drugs only in case of inefficacy or intolerance [60].

In the present paper, we thoroughly scrutinized the concept of progesterone resistance as an explanation for why 33% of patients do not respond to OCPs and progestins, with this percentage climbing even higher in women with deep nodular endometriosis [46,47,56,58]. Moreover, as mentioned earlier, there is a need for further treatment options and, as recently as 2020, several papers reported results from clinical trials on the two oral GnRH antagonists linzagolix and relugolix. These two studies clearly confirmed that GnRH antagonist suppresses ovarian function in a dose-dependent manner, allowing modulation of E2 levels which, according to the threshold hypothesis [86], may provide relief from endometriosis-associated pain while reducing side effects caused by extreme hypoestrogenism. We would advocate a strategy based on the different phenotypes of endometriosis, clearly recognized as three separate entities since the original publication by Nisolle and Donnez in 1997 [2]. Research on this strategy should be initiated, as taking these phenotypes into account would allow clinicians to discriminate between lesions from a clinical perspective.

Appropriate counseling of patients is of fundamental importance. It is the responsibility of health care providers to offer a comprehensive overview of the efficacy and side effects of all available therapies. The ideal treatment should be tailored to each individual woman according to the most troublesome symptoms (pain or infertility) and the phenotype of the disease. Furthermore, long-term adherence to treatment is a key issue and, in this context, efficacy and side effects are the most consequential points to consider. Indeed, the first goal of medical therapy is to be effective and avoid unnecessary surgical intervention. Avoiding repeat surgery in case of recurrence of pain is even more important, as it is well known that this is often the source of severe complications [123].

We cannot, of course, overlook the cost-effectiveness of medical management of endometriosis. Costs linked to endometriosis are estimated at $69.4 billion per year [124], so it is high time to promote and encourage further research, investigate innovations in treatment options and improve women’s access to quality care. Moreover, according to Wang et al. [125], the two recent FDA-approved doses of elagolix for the management of moderate-to-severe pain associated with endometriosis, namely, 24 months of 150 mg once daily or six months of 200 mg twice daily, both proved cost-effective versus leuprolide acetate over 1–2 years. Although further investigations, including assessment of efficacy and safety in real-world populations, potential for use of add-back therapy, and comparisons with OCPs and progestins are needed, we agree with Leyland et al. [126] that robust clinical evidence shows oral GnRH antagonists to be effective and well tolerated in patients with moderate-to-severe endometriosis-associated pain.

In conclusion, the title of a recently published expert review by As-Sanie et al. [127] appears to summarize the situation perfectly: “Assessing research gaps and unmet needs in endometriosis.”

We need to act sooner rather than later.

## Figures and Tables

**Table 1:** Efficacy and side effect of different doses of GnRH antagonist at 24 weeks.

	Elagolix				Linzagolix			Relugolix	
Assessments	150 mg Elaris-I	200 mg Elaris-I	150 mg Elaris-II	200 mg Elaris-II	75 mg	100 mg	200 mg	40 mg CT Spirit-1	40 mg CT Spirit-2
Pelvic Pain (OPP)	-	-	-	-	70.8	66.7	77.3	-	-
Dysmenorrhea (% responders)	42.1	75.3	46.2	76.9	58.3	82.1	84.1	75.5	75.2
NMPP (% responders)	45.7	62.1	51.6	62.2	72.9	64.1	72.7	58.5	66
BMD loss lumbar spine (%)	−0.32	−2.41	−0.72	−2.49	−0.80	−1.37	−2.60	−0.70	−0.78
Hot flushes %	23.7	42.3	22.6	47.6	19.0	28.8	45.6	10.4	13.6

Elagolix 150 mg once daily; elagolix 200 mg twice daily; linzagolix 75 mg, 100 mg and 200 mg once daily; and relugolix 40 mg plus add-back therapy once daily. OPP: overall pelvic pain; NMPP: non-menstrual pelvic pain.

**Table 2:** Efficacy and side effects of different doses of GnRH antagonist at 52 weeks

	Elagolix				Linzagolix			Relugolix	
Assessments	150 mg Elaris-III	200 mg Elaris-III	150 mg Elaris-IV	200 mg Elaris-IV	75 mg	100 mg	200 mg	40 mg CT Spirit-1	40 mg CT Spirit-2
Pelvic Pain (OPP)	-	-	-	-	69.2	53.8	82.4	-	-
Dysmenorrhea (% responders)	52.1	78.1	50.8	75.9	69.2	69.2	64.7	-	-
NMPP (% responders)	67.8	69.1	66.4	67.2	69.2	53.8	76.5	-	-
BMD loss lumbar spine (%)	−0.63	−3.60	−1.10	−3.91	−1.14	−1.40	−2.19	-	-
Hot flushes %	44	72	36	77	22	27	60	-	-

Elagolix 150 mg once daily, elagolix 200 mg twice daily, linzagolix 75 mg, 100 mg and 200 mg once daily). Patients randomized to linzagolix 200 mg were switched to linzagolix 100 mg at week 24.
